# Identity Negative Priming: A Phenomenon of Perception, Recognition or Selection?

**DOI:** 10.1371/journal.pone.0032946

**Published:** 2012-03-12

**Authors:** Hecke Schrobsdorff, Matthias Ihrke, Jörg Behrendt, J. Michael Herrmann, Marcus Hasselhorn

**Affiliations:** 1 Bernstein Center for Computational Neuroscience, Göttingen, Germany; 2 Max-Planck-Institute for Dynamics and Self-Organization, Göttingen, Germany; 3 Institute for Nonlinear Dynamics, University of Göttingen, Göttingen, Germany; 4 Georg-Elias-Müller Institute for Psychology, University of Göttingen, Göttingen, Germany; 5 Institute for Perception, Action and Behaviour, University of Edinburgh, Edinburgh, United Kingdom; 6 German Institute for International Educational Research (DIPF), Frankfurt/Main, Germany; University of Leicester, United Kingdom

## Abstract

The present study addresses the problem whether negative priming (NP) is due to information processing in perception, recognition or selection. We argue that most NP studies confound priming and perceptual similarity of prime-probe episodes and implement a color-switch paradigm in order to resolve the issue. In a series of three identity negative priming experiments with verbal naming response, we determined when NP and positive priming (PP) occur during a trial. The first experiment assessed the impact of target color on priming effects. It consisted of two blocks, each with a different fixed target color. With respect to target color no differential priming effects were found. In Experiment 2 the target color was indicated by a cue for each trial. Here we resolved the confounding of perceptual similarity and priming condition. In trials with coinciding colors for prime and probe, we found priming effects similar to Experiment 1. However, trials with a target color switch showed such effects only in trials with role-reversal (distractor-to-target or target-to-distractor), whereas the positive priming (PP) effect in the target-repetition trials disappeared. Finally, Experiment 3 split trial processing into two phases by presenting the trial-wise color cue only after the stimulus objects had been recognized. We found recognition in every priming condition to be faster than in control trials. We were hence led to the conclusion that PP is strongly affected by perception, in contrast to NP which emerges during selection, i.e., the two effects cannot be explained by a single mechanism.

## Introduction

Selective attention is the process of extracting behaviorally relevant information from the environment which provides us with a permanent stream of sensory input. Successful processing of a stimulus involves the act of focusing on the relevant as well as ignoring the irrelevant information. Contradicting the early hypothesis that attending is active and ignoring is passive, the active nature of ignoring has been revealed experimentally [1]. Subjects had to process lists of colored words in a standard Stroop task where the stimulus cards were ordered such that the ignored meaning of a color word always became the color in which the next word was written and which was to be named. People were slower in responding to those lists compared to unrelated ink colors and color names. Even if the semantic meaning of the color words has been ignored to fulfill the task, it must have entered the cognitive system. These results showed that stimulus selection can be assessed by systematic variation of distracting information. An important approach to investigate the processing of distracting stimuli is provided by the so-called negative priming (NP) paradigm where always a pair of consecutive tasks, called the prime and probe trial, are considered. Those tasks present a relevant and irrelevant stimulus – the target and distractor – and require a response to the target. NP manifests in a slowdown of the reaction in response to a probe target that was presented as the prime distractor. NP is considered a well suited approach to assess the selective aspect of attentional processing, since the ignored stimuli can be shown to be actively processed [2], [3]. Usually, NP is contrasted with the positive priming (PP) effect, a response-facilitation, which is observed when a target from the prime trial is repeated as target in the subsequent trial, the probe.

The NP effect has been observed in a wide variety of experimental contexts and therefore is a reliable and general phenomenon, for reviews see [4], [5]. In spite of this apparent robustness, many factors have been identified that can modulate, cancel or even reverse priming effects, e.g., the response stimulus interval [6]–[8], absence [7], [9]–[12] or saliency of the probe distractor [13], [14], task instructions [15], age [16]–[18], sex [19], perceptual load [4], [15], [20], composition of trials [21], [22], stimulus presentation time [23], stimulus onset asynchrony [10] and prime awareness [24]. The complexity of the phenomenon is the reason for many different theoretical accounts that have been formulated over the years, e.g. [3], [8], [25]–[30].

Historically, the most influential explanation of NP is distractor inhibition theory, which assumes irrelevant stimulus representations are actively suppressed, thereby supporting the selection of the relevant target stimulus [3], [14], [27], [31]. Inhibition of the cognitive representation of the distractor is postulated to persist for some time which produces the NP effect. Distractor inhibition theory has also been expressed in terms of a dynamical model: When perceptual input is no longer present, persisting inhibition drives the distractor representation below a baseline activation level. The NP effect directly results from the time the probe target representation activation needs to reach baseline activation again. Distractor inhibition assumes selection to operate on a semantic or postcategorial level [2] and it can thus also explain findings that report NP in semantic priming tasks, e.g. [32]. The slowdown of the reaction in the probe trial can thus be regarded as a direct correlate of the amount of inhibition in the prime display. Distractor inhibition theory was developed in numerous contributions over the years [2], [3], [14], [31], [33]–[37].

Nevertheless, there is a growing consensus in the literature on identity negative priming that NP is primarily a memory phenomenon, i.e., it is better explained by effects stemming from memory retrieval rather than of distractor inhibition. Retrieval theories originate from Logan's instance theory of automatization, which states that the processing of similar successive trials leads to a high level of automatization and optimization [38]. One optimizing strategy is a retrieval of the previous episode from memory whenever stimuli or even parts of it are repeated. Simultaneously, a slower, algorithmic processing is carried out which can interfere with the retrieved information. Episodic retrieval theory [8], assumes the distractor object to be stored in conjunction with the directive not to respond to it. A perceptual similarity of prime and probe stimuli, e.g. if an object is repeated, triggers an incidental retrieval of the prime episode. The associated inhibitory directive is recalled as well. In case of congruent instructions as in the case of a target repetition condition, the retrieval is beneficial, whereas in distractor-target trials incongruent directives interfere, leading to a slowdown of the reaction by an inhibitory after-effect. The original theory was modified several times, with more recent retrieval accounts stressing the point that prime retrieval reinstates processing operations that have been carried out during the prime episode [39]–[41]. A recent descendant of episodic retrieval theory is response retrieval theory which assumes that only the prime response is recalled in case of a perceptual match between prime and probe [29]. In that context, the confounding of response repetition and priming condition was nicely accounted for.

Evidence in the form of results favoring either one or another theory of NP has accumulated that a combination of distinct mechanisms might be responsible for the NP effect, e.g. [14], [25], [41]. These integrative accounts agree that there are at least two more or less independent mechanisms that are responsible for NP, persisting inhibition, e.g. [3], and memory retrieval, e.g. [8], [39].

Although retrieval processes can account for NP effects, they form only a subordinate part in the selection of a target against a distractor. Additional, possibly inhibitory processes are necessary to select information for goal-directed behavior, which are vaguely described as slow algorithmic processes in the context of the retrieval theories. Because different processes may interact, it is difficult to distinguish them by means of behavioral measures alone [42]. Furthermore the contributions of inhibitory and retrieval processes might vary considerably depending on subtle differences between paradigms. For example, it has been found in several studies that NP may depend on contextual conditions and other experimental factors [14], [43]. On the contrary, a meta analysis of age-related NP experiments did not find evidence for the effect that certain paradigms differ significantly in the observed priming patterns [16].

Studies investigating the NP effect usually aimed at supporting or rejecting one of the explanatory accounts of NP, e.g. [8], [37]. The focus of the current study is on the identification and localization of the cognitive processes during trial processing which are related to the interference observable as NP. More specifically, we argue that trial processing consists of distinct operations that, at least to some degree, are executed in sequential order: First, the stimuli are perceived by the visual system; shape, color and other feature information is extracted. At some stage during perception and recognition, a target-selecting process comes into play that modulates the processing of relevant and irrelevant information. Presumably, these top-down processes operate on early perceptual representations as well as on higher semantic levels [44], [45]. Finally, once the target has been identified, the correct response must be initiated. At some point during all these parts of trial processing, the distractor representation is treated differently from the target representation. The NP effect shows that the distractor is not perceptually blocked in the beginning (early selection), but recognized and also memorized. Target and distractor are therefore processed in parallel for a certain part of a trial, and at some point the two representations are processed differently according to their role as distractor or target, respectively. We believe that it is crucial for the further investigation of the NP effect to precisely determine its temporal localization relative to certain aspects of trial processing. Even though the brain is computing massively in parallel, seriality is an inherent feature of many brain processes, see e.g. [46]. In the current study we build on the assumption that some processes can not start to operate efficiently until other processes have reached a certain degree of maturity.

There have been various attempts to assign NP to a particular process, each of which addressed a single aspect. Tipper and Driver showed that NP is also observable if only a semantic relation between prime distractor and probe target is given [32]. They concluded that NP cannot be a perceptual phenomenon. Grison and Strayer found a minor influence of perceptual manipulations on NP, thus arguing in the same direction [13]. In addition, May et al. concluded in their review of several studies where perceptual features and response modalities were manipulated that NP is produced at an abstract level [25]. But they stated that the considered studies all used semantic material, thus the conclusion that NP acts on a semantic level may be an over-generalization due to the specific paradigm used. Therefore, May et al. stated that a larger basis of data is necessary in order to answer the question at which level of processing NP is produced [25]. In a recent ERP study we found comparable processing of distractor-target and target repetition trials in the early stages of a trial and diverging processing at later stages associated with activation of higher brain regions [47]. This pattern supports the following propositions: First, object repetition may lead to a faster perceptual processing for both NP and PP conditions; second, a delaying, negative priming effect occurs after the full categorization of the stimuli, which is in contrast to predictions derived from distractor inhibition theory. Thus, the acceleration which is characteristic for PP appears to be produced by early processes different from the later ones that seem to be responsible for NP. This result questions the discussion of NP and PP as two different byproducts of the same mechanisms.

To separate perceptual effects from those that occur later during target selection by purely behavioral measures, we resolve a confound present in most NP studies: the determination of an object being target or distractor is usually made according to a fixed property (e.g., the color green). Such a paradigm directly entails a greater perceptual dissimilarity for the NP condition as compared to the PP condition where the target is repeated identically. Comparing NP and PP with the control condition therefore cumulates the impact of the switch of the target feature and the distractor-to-target manipulation. To resolve this conflict, we introduce a switch of the effective target feature in individual trials. This simple experimental manipulation allows to orthogonally vary perceptual prime-probe similarity independently of priming condition: There are both NP and PP conditions with identical and dissimilar displays.

A switch of the target color could be interpreted as a task switch. There is a strong basis of literature on task switching and interference, for reviews see [48], [49]. However, the focus is on interfering task sets, and not on interfering stimuli. Up to our knowledge, only very few studies considered task switching in conjunction with NP, see [50]. Implementing a picture naming and a word reading task with stimuli containing both a word and a picture, Waszak et al. considered priming effects over several intervening trials whenever a stimulus has appeared before in any display [50]. Interferences from two successive trials were not considered. In comparison, our switching of target color appears to be a relatively weak manipulation and therefore we expect little or no effect. In contrast to task switching studies where the subject had to perform two distinct tasks (e.g. picture naming vs. word reading), the instruction in our case was identical in all cases: ‘respond to the item shown in the given color’.

A switching of the target feature creating identical prime distractor probe target pairs was previously implemented in several studies to compare feature mismatch theory and episodic retrieval, see e.g. [51]. Furthermore, Tipper implemented a switch in the target color from red to green in all prime-probe pairs throughout one experimental block (i.e., all NP conditions showed an identical prime distractor and probe target) and found a substantial NP effect [3]. A more complex picture was drawn by Milliken, Tipper, and Weaver, who found NP occasionally with no clear dependence on the way of cuing a trial [52]. MacLeod et al. used words as stimuli and changed target color within every prime-probe pair [51]. NP was only observed in trials without a target color switch. In case of the prime distractor having the same color as the probe target, they found a facilitating effect. One aspect is common to all the above studies: The switch occurred regularly, thus predictably. Another important aspect is the lack of trials that show the repeated object as probe distractor. Therefore, a strong bias exists to attend both prime target and prime distractor, as they are very often repeated as the probe target and never as the probe distractor. We will investigate both aspects in the current study.

We applied the target-feature manipulation in a series of three experiments implementing a classical voicekey identity negative priming paradigm [3],[30]. Experiment 1 changes the color that identifies the target between blocks rather than between trials to assess the general impact of using different colors for target-selection. This approach allows to identify for a potential interaction of target color and priming effects in the following experiments [14]. In Experiment 2 we implemented an unpredictable target color switch on a single trial basis by showing a color word directly before the stimuli are presented. This manipulation destroys the confounding of the repeated object being shown in the same or a different color and the priming conditions as argued above. Finally, in Experiment 3 the order of stimuli and color cue was reversed, thus artificially separating the early phase of trial processing, i.e., stimulus perception and recognition from the categorization and response generation phase. This is because a stimulus object can be identified as target or distractor only after the color cue has been shown. The main question of Experiment 3 is therefore the presence or absence of NP in the early processing stage.

All three experiments implemented a comprehensive set of stimulus conditions, implementing all possibilities of single-object repetitions. We label the experimental conditions according to Christie and Klein: control (no repetition), distractor-to-target (DT), target-to-distractor (TD), target repetition (TT) and distractor repetition (DD) [53]. Such a condition set results in an unbiased presentation of stimuli and conditions: Consider the case when only DT and TT trials are presented. In this case, the repeated object is always the to-be-attended probe target, which could bias the subjects to make additional effort to keep track of both items. Adding DD and TD trials to the condition set balances the repeated object between being relevant and irrelevant in the probe trial. Additionally, the design of Experiment 3 requires the subject to attend to both stimuli in the same way before the target color is announced. If only DT and TT trials were presented, the subjects would know the role of the repeated object in the probe right away. The inclusion of all conditions destroys the bias such that no information about the probe role of the objects is available in advance. Finally, the two complementary conditions, TD and DD, are rarely reported in the literature. This is in contrast to their potential to assess the validity of any theory on NP because most theories are general enough to derive predictions also for these conditions.

## Experiment 1

Experiment 1 was a baseline experiment designed to investigate priming-specific effects of the two different target colors we will use in the following experiments. This was a necessary prerequisite for our main research question which required a target-color switch on a per-trial basis. There are empirical indications for incidental features (such as color) to act as moderating factors on priming effects. One finding is that the NP effect may increase with growing saliency of the distractors [13], [37], [54].

With regard to Experiments 2 and 3, where a switch of the target color always entails a switch of the distractor color, Experiment 1 was intended to rule out that the target color has a moderating effect on priming. Such a modulation could be accounted for in the analysis of the subsequent experiments using the results from Experiment 1, i.e., determining a correction term to be applied when pooling responses to red and green targets, respectively. Even though Experiment 1 was basically a replication of former identity negative priming experiments, see e.g. [3], [30], [55], we present it in full length in order to introduce the baseline priming effects we expect in our setting. Experiment 1 consisted of two blocks of trials, one of which implemented green as the target color, the other one requiring identification and naming of the red object.

We expected a replication of priming effects usually found in identity NP studies with visual stimuli and a naming response, see e.g. [22], [47]: While TT trials usually cause a strong acceleration (PP), the acceleration is weaker in DD trials. DT and TD trials show a deceleration (NP) which is often weaker for TD trials. Reaction times in response to green targets were expected to be longer than in response to red targets due to the higher saliency of the color red as compared to green. Based on studies on the impact of distractor saliency on priming as well as our own simulations using an adaptive-threshold model of NP [30], we expected the DT and TD effect to be stronger for red distractors than for green ones, and the acceleration of TT and DD trials to be weaker with green targets.

### Method

#### Participants

Thirty young adults (19 female, 11 male) took part in the study (M = 24.5 years, SD = 1.6 years). The participants were rewarded with course credits or paid 15 . All subjects had normal or corrected-to-normal vision and no color discrimination disabilities. They were not informed about the specific purpose of the experiment and had not taken part in a previous study employing similar stimulus material. The study was reviewed and approved by the Ethics Committee of the MPI for Dynamics and Self-Organization, Göttingen. The Committee did not require that informed consent was given for the experiment: voluntary participation in the task was accepted as implied consent.

#### Materials

Stimuli were six different objects, represented by hand-drawn pictograms that either were shown in green or in red color. The stimuli were designed to possess a number of desirable qualities such as a comparable visual complexity and spatial coverage. The comparability of the objects was validated in pilot studies and the stimuli were already successfully used in some NP studies [22], [30], [56]. As the modality of response, we used voice recording together with a sound level threshold to determine the reaction time for every trial. We therefore chose object labels that commence with a plosive and consist of a single syllable for a sharp onset of the sound signal. The experiment was conducted in German language and the corresponding labels were *Bus*, *Ball*, *Baum*, *Buch*, *Bett*, *Bank* (bus, ball, tree, book, bed, bench).

#### Design

We implemented a 5 (priming: CO, DT, TT, TD, DD)
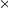
2 (target color: red, green) design. Both factors were varied within-subject. Target color was kept constant during blocks of 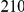 trials each and blocks were presented in random order across subjects. Object presentation was balanced across the different priming conditions as well as their appearance as target or distractor using a software designed for avoiding sequence structure [57]: Each of the pictograms appeared an equal number of times (both as target and distractor) and the number of trials was counterbalanced across the stimulus-repetition conditions.

#### Procedure

The experiment was conducted in a special chamber optimized for low noise and standardized lighting conditions. Participants were tested individually in sessions that lasted no longer than one hour. Before the start of the experiment, the line drawings of the experimental stimuli along with their names were presented in a neutral black coloring. The subjects were told that in each trial they would see two of these objects overlapping one another, one drawn in green and the other one drawn in red. Participants were instructed to name the target objects as quickly and correctly as possible while ignoring the superimposed distractor object. To familiarize participants with the experimental procedure, 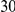 practice trials preceded the main session.

**Figure 1:**
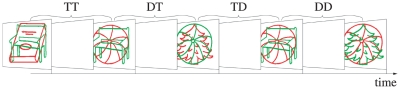
An example sequence of stimuli of **Experiment 1** **in the part with target color green.** All realized experimental conditions are shown except the control condition, i.e., unrelated objects in prime and probe. The fixation cross is omitted in the shown sequence for clarity.

Experiment 1 consisted of two parts of 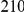 trials each, subdivided into 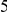 blocks of 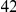 trials. After each block, subjects were allowed to take a short break. In a single trial, subjects were shown the following series of displays: (1) a fixation cross, centered on the screen for 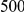 ms, (2) a display containing two superimposed objects in the focal area until the subject responded but no longer than 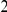 seconds and (3) a blank screen for a randomized duration drawn uniformly from the interval between 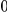 and 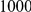 ms, as this procedure has proven in our lab to produce pronounced priming effects. The effective response-to-stimulus interval (RSI) was therefore a random duration between 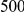 ms and 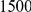 ms. An exemplary sequence of displays of four trials requiring a response to green objects is shown in Fig. 1. Behavioral errors were noted by the experimenters when subjects failed to give the correct answer.

#### Outlier correction

All reaction times from trials in which a behavioral error occurred were excluded from the analysis together with the immediate successor. Reaction times below 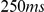, above 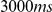 and those where the difference to the mean of the experimental condition of the particular subject exceeded two times the standard deviation were also excluded. Finally, the first two trials of each block of 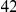 trials were removed from the dataset to avoid the inclusion of transient effects when the subjects restart the task after a break. In summary, not more than 10% of trials per condition and subject were excluded from the analysis.

### Results

#### Reaction times

The repeated-measures ANOVA on target color (red, green)
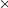
priming (CO, DT, TT, TD, DD) yielded main effects for color, 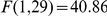, 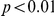, and for priming, 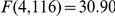, 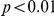, but no interaction between the two, 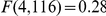, 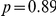. All four priming conditions show priming effects in the expected directions (DT: 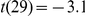, 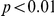; TT: 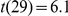, 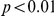; TD: 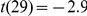, 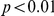; DD: 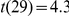, 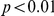), see also Table 1. Throughout the paper, all 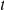-tests were corrected for multiple comparisons using Holm's method [58]. According to the main effect in the above ANOVA, responses to red targets, 709 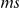 (sd = 95 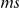), are faster than responses to green targets, 758 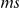 (sd = 89 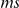).

**Table 1:** Summary of results of Experiment 1.

	Target color			
	Red		Green	
Condition	Mean RT [ 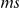 ]^a^	Error rate [%]^a^	Mean RT [ 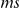 ]^a^	Error rate [%]^a^
CO	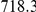 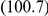	2.1 (3.3)	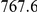 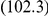	1.4 (2.2)
DT	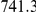 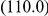	2.8 (2.5)	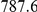 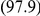	2.2 (2.9)
TT	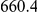 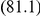	1.4 (2.4)	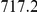 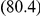	1.6 (2.4)
TD	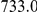 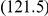	1.9 (2.3)	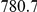 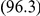	2.2 (2.2)
DD	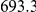 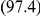	2.2 (2.6)	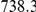 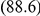	2.3 (3.1)

a Standard deviation in parentheses.

b Difference of CO and priming condition.

### Error rates

The two-way ANOVA on error rates with factors target color (red, green)
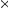
priming (CO, DT, TT, TD, DD) yielded no main effect of color 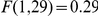. The main effect of priming just missed significance 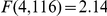, 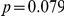. The interaction between the two was not significant, 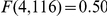. Generally, the error rates were too low to yield any significant results.

### Discussion

Experiment 1 produced the expected priming effects for all conditions: Independently of target color, we found a response facilitation in TT and DD trials and a deceleration in DT and TD trials. Furthermore, we observed a main effect of target color, indicating that reactions to green targets were slower than reactions to red targets. There was no interaction between target color and priming.

Priming effects in the DT and TT condition are a replication of many existing studies, e.g. [3]. It has been shown that negative priming may depend on the composition of the trial sequence, e.g. [3], [7], [9], [10], [55]. It is therefore interesting to note that the classical effects also occur in a setting where additional priming conditions (DD/TD) have been realized. The results from TD and DD conditions are also in accordance with previous investigations [56]. The shorter reaction times for red targets are presumably due to the higher saliency of the color red. The higher saliency apparently helps to guide attention to the target which facilitates recognition and categorization [59].

The strong priming effects found in Experiment 1 proved the suitability of the voicekey paradigm for the issue addressed in this paper. The missing interaction of target color and priming facilitates the analysis of the subsequent experiments. Because of the missing interaction, it is not necessary to consider target color as a factor in the other experiments which mix trials with red and green targets on a per-trial basis.

## Experiment 2

**Figure 2:**
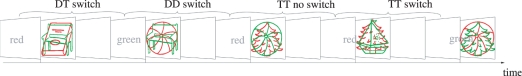
An example sequence of stimuli of **Experiment 2** **.** A trial begins with the display of a grey color word, followed by the actual stimuli. The fixation cross is omitted in the shown sequence for clarity.

Experiment 2 was designed to assign the reaction-time effects in the priming conditions to perceptual processing or possible interferences during later processing steps, i.e., target selection and response generation. Extending Experiment 1, we introduced a trial-wise target-color switch in the form of a color word indicating the target color in the current trial (see Fig. 2). Besides the color switch on a per-trial basis, the experimental procedure was identical to that of Experiment 1. As argued above, the color switch resolved the confounding of priming and perceptual similarity, i.e., whether an object was repeated in the same color or not. For example, we realized TT trials where the repeated target was shown in a different color in prime and probe even though color was the feature used for target selection.

Retrieval theories explain the NP effect in terms of memory processes. They suggest that memory retrieval is initiated depending on the degree of similarity of prime and probe stimulus and so causes interference in case of a mismatch [8]. Both episodic retrieval and response retrieval theory thus postulate that the NP effect predominantly occurs in the later phases of trial-processing: at least the perceptual processes have to reach a certain stage such that a match of the current percept and the memorized episode can be determined. While original episodic retrieval attributes the effect to a mismatch between the entire prime and probe episodes, response-retrieval theory postulates an interference only between actual and retrieved probe response [29]. Since retrieval of episodes is said to depend on the degree of similarity between the two displays, priming effects are modulated by the perceptual change of the repeated object.

In contrast to the overall priming effect, the above observation implies that a better match leads to a stronger effect, because already the pure repetition of an object is said to trigger the retrieval of the prime episode [38]. Equivalently, retrieval theories expect an acceleration of the response for TT trials in both no-switch and in switch conditions due to the supporting content of the episode (or response) retrieved from the prime trial. Minor changes in the size of the priming effects are expected to be caused by the different perceptual similarity: If the percepts are identical, the search in memory for the stored episode might be faster and more accurate compared to identical objects that differ in color.

In contrast, distractor inhibition theory assigns NP to an impaired semantic recognition of the repeated object due to persisting inhibition in the case that the object was presented as prime distractor [3]. Distractor inhibition theory assumes that inhibition occurs during the recognition process, which consists of perception and semantic categorization. However, because inhibition is assumed to operate on a semantic, cognitive representation of the objects, no dependency of the NP effect on the switch of target color is predicted. The distractor inhibition theory postulates a residual activation of the semantic representation of the prime target and thus predicts an acceleration in TT switch trials.

Both of the two prominent NP theories explain negative and positive priming in the same conceptual framework. In contrast, evidence from a recent event-related potential (ERP) study by Behrendt et al. suggests that NP and PP are caused by different cognitive processes [47]. According to their results, positive priming as it occurs in traditional TT trials is a perceptual phenomenon because priming correlates, i.e., differences to the ERP of a control trial, occur in early, perceptual stages of the ERP. For DT trials, the early EEG reflecting perceptual processes is similar to the one in TT trials, indicating an easier recognition due to stimulus repetition. Only on a later processing stage the ERPs of both conditions diverge. This result indicates that the deceleration in DT trials is not a perceptual phenomenon but rather happens later, during semantic processing.

From these considerations, we derive predictions for Experiment 2. Following distractor inhibition theory, NP should be independent of perceptual similarity, whereas episodic retrieval predicts a stronger effect for identically repeated items. The situation is less clear for positive priming. In accordance with the theories, we expect a deceleration in DT trials to occur both in switch and in no-switch trials because they are caused by processes at a conceptual level. Conversely, based on the study by Behrendt et al., we expect the acceleration in no-switch TT trials to vanish in trials implementing a switch of the target color [47]. This prediction is due to the attribution of positive priming to an early, perceptual stage of trial processing. For the TD and DD condition, we do not have a dedicated reference attributing it either to perceptual or to conceptual processing. We therefore argue tentatively, based on the empirical experience with TD and DD conditions [56], [60], that TD will produce similar effects as the DT condition and that DD will produce effects that are comparable to the TT condition. Reaction times in DD trials were assumed to benefit from a facilitated figure-ground separation which is produced by the afterimage of the distractor from the prime trial [56].

### Method

#### Participants

Experiment 1 and Experiment 2 were run in a single session, thus the subjects in both experiments were identical.

#### Materials

Besides the color-cue (“rot” (red) or “grün” (green)) displayed in grey and centered on the screen to indicate the current target color, the displays were identical to the ones in Experiment 1.

#### Design

A 5 (priming: CO, DT, TT, TD, DD)
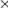
2 (color switch: switch, no-switch) design was realized where both factors were varied within-subject. Note that the stimulus displays were identical for pairs of priming
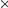
switch conditions. The following conditions had an identical stimulus display: DT & DD switch, TT & TD switch, TD & TT switch and DD & DT switch.

Particularly, trials that did not require a color switch differed from trials in Experiment 1 only in the presence of the color cue preceding the stimuli, see Fig. 2. The effective response stimulus interval was longer as compared to Experiment 1, because the subject's processing of the cue added to it.

#### Procedure

Experiment 2 consisted of 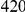 trials with breaks every 42 trials. We implemented a standard way of unpredictable task switches by pseudo-randomly presenting a color cue, i.e., a color word presented in grey, indicating the target color for each trial. The presentation of the color cue in a neutral color avoided possible cue mismatch influences, e.g. [61]. The color cue was removed after a button press by the subject and the actual trial was started, see Fig. 2.

### Results

#### Reaction times

All reaction times and error rates are summarized in Table 2. The two-way ANOVA with factors color switch (switch, no switch)
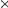
priming (CO, DT, TT, TD, DD) yielded significant main effects for switch, 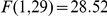, 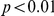, and for priming, 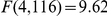, 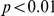, and a tendency for a significant switch by priming interaction, 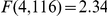, 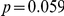. Thus, trials that followed a target color switch, 822 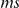 (sd = 129 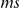), were slower than trials not requiring a color switch, 805 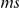 (sd = 118 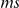). In order to further investigate the origin of the tendencial interaction, we ran separate ANOVAs for the different priming conditions.

**Table 2:** Summary of results of Experiment 2.

	Color repetition		Color switch	
Condition	Mean RT [ms]^a^	Error rate [%]^a^	Mean RT [ms]^a^	Error rate [%]^a^
CO	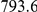 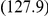	2.0 (2.8)	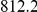 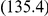	2.2 (3.2)
DT	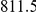 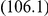	2.1 (2.0)	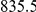 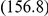	2.0 (1.9)
TT	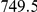 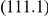	1.9 (2.2)	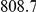 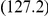	2.1 (2.8)
TD	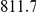 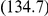	1.6 (2.2)	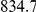 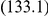	1.8 (2.1)
DD	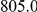 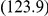	1.8 (2.4)	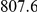 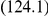	2.8 (3.3)

a Standard deviation in parentheses.

b Difference of CO and priming condition.

The ANOVA switch
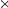
(CO, DT) yielded tendencial main effects for switch, 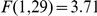, 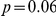, and for priming, 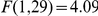, 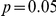, and no significant interaction between the two, 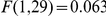. DT was slower than CO (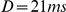).

The ANOVA switch
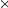
(CO, TT) yielded main effects for switch, 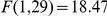, 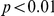, and for priming, 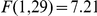, 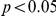, as well as a significant interaction between the two, 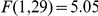, 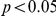. Detailed 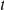-tests showed that in the no-switch case TT was faster (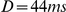) than CO, 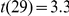, 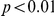, whereas for color switch trials TT was not significantly different from CO, 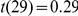, 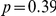.

The ANOVA switch
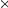
(CO, TD) yielded main effects for switch, 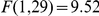, 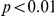, and for priming, 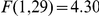, 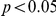, but no significant interaction between the two, 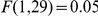. TD was slower than CO (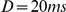). The ANOVA switch
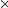
(CO, DD) did not yield any significant effects.

#### Error rates

The two-way ANOVA on error rates with factors color switch (switch, no switch)
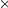
priming (CO, DT, TT, TD, DD) yielded neither significant main effects for switch 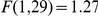 or color 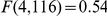, nor an interaction between the two 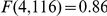. Again, the error rates were too low to yield any interpretable results, see Table 2.

### Discussion

Results were consistent with our hypotheses: In color repetition trials priming effects were similar to those found in Experiment 1, and the introduction of the switch affected the priming effects as predicted. While the effects in the DT and TD conditions were independent of the color switch, they disappeared in the TT condition when the target color was switched. Color switch trials were slower than color repetition trials.

The longer response-stimulus interval (RSI) compared to Experiment 1 could have influenced priming effects [6]. However, the only difference between the results of Experiment 1 and color repetition trials in Experiment 2 was that the acceleration in DD trials vanished. Therefore, the impact of the prolonged RSI seemed to be negligible in the present voicekey paradigm in the other three conditions. Switch trials were slower than those which repeated the target color. The general slowdown can be attributed to the updating of the task set [62] whenever a probe color cue was presented that was different to the prime target color. Thus the subjects had to adjust the stimulus processing accordingly, which led to the observed effects.

According to the findings of Experiment 1 and previous studies [29], [56], [63], we expected an acceleration in DD trials, which could be explained by a figure-ground separation effect. In contrast, in Experiment 2, we found no priming effect for DD in no-switch trials, which can be explained by the destruction of the afterimage by the color cue which might have effectively operated as a masking stimulus [64].

Priming effects in DT and TD conditions were independent of a color switch. This could either reflect opposing influences, e.g. a speeded recognition of the identically presented prime distractor and a more pronounced slowing due to the stronger retrieval, or, in agreement with EEG correlates [47] the fact that perceptual processes play a minor role in the slowdown of reactions in DT and TD trials. Negative interference apparently happens on a semantic level of processing or later during response generation.

Only priming in the TT condition showed a dependency on the color switch. When no switch was required, classical positive priming was observed, i.e., TT trials were much faster than CO trials. However, in case the target color switched and the perceptual identity was thus destroyed, the positive priming effect disappeared. The degree of perceptual match of the target apparently has a large modulating effect on PP. This might be taken as further evidence for the position that if perceptual processes do not directly account for PP, they at least form a window towards those facilitatory effects.

Although the identical repetition of the prime target in a TT no-switch trial confounds with an identical target color cue, cueing both the prime and probe trial, we think it is rather unlikely that the target-cue was integrated into episodic memory along with the target and distractor stimuli. Spatiotemporal proximity has often been demonstrated as necessary for feature-binding [65]. In fact, it is a necessary prerequisite for binding that stimuli be perceived as “belonging to one another” in a perceptualgrouping sense [66]. Certainly, this was not the case in our setup: The target cue appeared temporally separated from the distractor and target stimuli.

An alternative explanation of the disappearing PP effect in TT switch trials would be the response repetition effect [67] which is a bias to respond differently in case of a change in the stimuli. Due to our confounding of experimental condition and response relation, see [29], i.e., responses are only repeated in TT trials, we cannot address this question within our paradigm. In constrast to the DD condition, PP is not sensitive to the masking effect by the color word. While the acceleration in DD trials seems to occur in an early perceptual phase, the attention attributed to the target makes the percept survive the color cue.

The conclusion that PP in TT conditions is sensitive to differences in perceptual processes implies that positive and negative priming are caused by different cognitive processes and that explaining both effects in the same theoretical framework might be an inadequate approach. This is reflected by the failure of both inhibition and retrieval theories to predict the missing acceleration in TT conditions for color-switch trials observed in our experiment. As a consequence, we suggest that future efforts to find explanations for NP should focus on the conditions that involve role-reversal (DT and TD). A limiting of the explanatory range of NP theories might help to finally converge at a generally accepted and simple theory of NP.

Experiment 2 indicates that NP is not a perceptual phenomenon, in fact, it provides a temporal allocation of NP effects to the late processing stages of a trial: The interference happens during semantic processing of the stimuli, in the interval from stimulus classification to response generation. These processes include semantic recognition of stimuli, selection of the target against the distractor and response selection. In order to explore the origins of NP in more detail, we carried out a third experiment providing a distinction of trial processing into recognition of stimuli and selection of the response.

## Experiment 3

**Figure 3:**
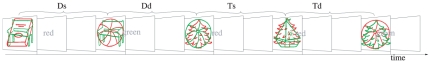
An example sequence of stimuli of **Experiment 3** **.** A trial begins with the display of the stimulus compound which is replaced by the color cue after a button press by the subjects. The fixation cross and the mask are omitted in the shown sequence for clarity.

In Experiment 2, we found NP to be independent from perceptual identity but rather is produced during later processing, i.e., target recognition until response generation. In these later stages, at least to some part, a sequential processing takes place: The stimuli have to be recognized before the response can be determined. To assess the temporal localization of NP in the later part, we slightly altered our paradigm. We realized a temporal separation of the two processes using a technically simple manipulation, the presentation of the target color cue after the stimulus objects had been identified, see Fig. 3. The subjects were instructed to indicate when they had finished the identification of the stimuli by a button press. The immediately following replacement of the stimuli by the color word triggered the response-selection.

Even though the question whether the objects are sufficiently recognized seems rather introspective, subjects easily solved the tradeoff between fast and correct responses. For efficient responding, subjects had to avoid an early button press which would have erased the stimuli and made a response impossible as well as a late button press which would unnessecarily prolong the response. Without the color cue, no information about the role of the objects as target or distractor was available to the subjects. We thereby ensured that object recognition and target and response selection were processed in a strictly sequential order.

The interpretation of the interferences with former episodes during target selection and response generation is not unambiguous. Therefore, we will focus on the perception and recognition part of a trial, i.e. from stimulus onset to the button press. We assume that the subjects were primed similarly as in Experiment 2 at the beginning of the probe trial: At the end of the prime trial, subjects had given the response and were thus likely to have had a strong mental representation of the target object even though it was not visually present. Also the intermediate color word is given in both experiments. We argue that, in order to select the target out of the stimulus compound, the memorized distractor had to be ignored similarly as in Experiments 1 and 2. Thus the difference between Experiment 2 and 3 lies exclusively in the processing of the perceptual phase: Both objects have to be attended to instead of just the current target.

For the RT in the recognition phase in our paradigm, distractor inhibition theory predicts a negative priming effect in trials where the distractor is repeated. This is independent of the color of the presented stimulus, as the semantic representation of the distractor is supposed to be inhibited [3]. Correspondingly, trials where the prime target is repeated should produce a positive priming effect because the prime target representation would still be activated. Clearly, in TT trials with repeated target color, a faster recognition is expected resulting from the higher perceptual match. In contrast, memory-based explanations of NP expect NP to happen at least to a considerable extent later in a trial [8]. Thus, a presence of NP in the early recognition phase would favor distractor inhibition theory. Conversely, an absence of NP in the early phase would favor retrieval theories.

Following the retrieval approaches, the findings from our ERP study [47], and on the basis of the results from Experiment 2, we expect an acceleration of the recognition phase in all trials where an object is repeated from prime to probe, i.e., all four priming conditions. In addition, when the stimulus is repeated identically the acceleration should be faster than for repetitions in a different color.

### Method

#### Participants

Twenty young adults (13 female, 7 male) were tested (M = 23.7 years, SD = 1.45 years). They received course credit or were paid 10 . All participants had normal or corrected-to-normal vision, no color discrimination disabilities and were naïve about the aims of the experiment. Again, the study was reviewed and approved by the Ethics Committee of the MPI for Dynamics and Self-Organization, Göttingen. The Committee did not require that informed consent was given for the experiment: voluntary participation in the task was accepted as implied consent.

#### Materials

The identification of stimuli before the target selection was much more demanding for the subjects as compared to Experiments 1 and 2. This led several subjects in a preliminary study to use an afterimage strategy which shifted part of the stimulus identification to the selection phase of the trial. This strategy interfered with our assumption of seriality. In order to enforce a serial trial processing, we introduced a mask between the stimulus presentation and the appearance of the target cue. The mask consisted of red and green dots at the location of the stimulus compound in a similar density. The effectiveness of the mask to destroy the afterimage was confirmed in a pilot study. All other stimuli were identical to the ones used in Experiment 2.

#### Design

Although the experimental manipulation between Experiment 2 and Experiment 3 is minimal, there is a difference in the design as we analyse only the recognition part of a trial. Our reaction time marker was the recorded button press of the subjects, which indicates a subjective recognition of both the green and the red object. This initiated the replacement of the two stimuli by the color word cue. The reaction time corresponding to that button press is interpreted as the time to recognize both objects and was subject to our analysis of Experiment 3. As in the early trial phase there is no knowledge about whether or not the target color was switched, therefore we do not have the same factorial design as in Experiment 2. The experimental conditions are defined by whether the repeated object is shown identically or in a different color in the probe trial. We label the conditions as Ts (target repetition in the same color), Td (target repetition in a different color), Ds (distractor repetition in the same color), Dd (distractor repetition in a different color).

#### Procedure

Experiment 3 only differed from Experiment 2 by the order in which the color cue and the stimulus objects were presented. The subjects were confronted with target and distractor in the beginning of a trial and had to press a button when they had recognized both stimulus objects. Between the stimulus objects and the target color cue, subjects saw a mask for 100 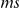 in order to erase any afterimage. The color cue was present until the subject responded.

### Results

#### Reaction times

All reaction times and error rates are summarized in Table 3. The one-way ANOVA on priming conditions (CO, Ts, Ds, Td, Dd) yielded a main effect for priming, 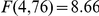, 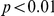. Reaction times showed an acceleration in Ts, 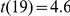, 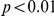, Td, 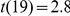, 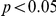, Ds, 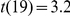, 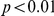, and Dd trials, 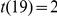, 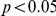. As expected, the acceleration effects in trials with identically repeated objects were stronger (D = 64 ms) than in trials with objects being repeated in a different color 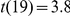, 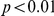. Note that the analysis is based only on the recognition reaction times.

**Table 3:** Summary of results of Experiment 3.

Condition	Mean RT [ms]^a^	Error rate [%]^a^
CO	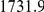 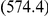	4.5 (3.4)
Dd	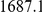 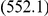	3.9 (3.2)
Ts	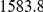 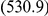	3.5 (2.9)
Td	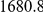 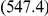	3.5 (1.6)
Ds	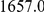 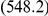	4.2 (2.4)

a Standard deviation in parentheses.

b Difference of CO and priming condition.

#### Error rates

The one-way ANOVA on priming conditions (CO, Ts, Ds, Td, Dd) yielded no main effect for priming, 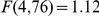 Still, error rates were too low to produce considerable effects, see Table 3.

### Discussion

The fact that a faster recognition of the stimulus objects was observable at all priming conditions points to perceptual benefits for repeated stimuli. An identical repetition of the prime target led to the fastest reaction. The accelerations of the recognition in all four priming conditions can originate either from a higher perceptual or semantic match. Episodic retrieval theory suggests memory retrieval to be modulated by the perceptual match, which can explain the difference of trials with a repetition in the same or in a different color [39]. The theory states that memory retrieval acts in parallel to a slower, direct computation of the appropriate response and facilitates responding once retrieval is completed [8]. This could be understood as a modulation at the stage of response selection. In our paradigm, response selection is not part of the recognition time and therefore, particularly response retrieval theory [29] cannot directly explain the recognition benefits we observed. A possible line of argumentation would be that both probe objects are attended like a target in the recognition phase, i.e., a selection of two out of the possible six responses takes place. In that case, episodic retrieval theory would postulate a reaction time modulation by retrieval of information from the prime trial. Nevertheless, in such a scenario the incongruent information in Ds and Dd trials should lead to a deteriorated response, which was not observed. The greater acceleration for prime target repetition could also be caused by a certain strategy to look for the prime target first.

Given that in the response phase the target has to be selected against the distractor, distractor inhibition theory assumes residual inhibition from the prime trial to produce NP by hampering the activation of the distractor representation [3]. Thus, distractor inhibition predicts a slowdown already in the recognition phase in Dd and Ds trials. Concerning PP the theory is not very explicit. Simulations of a neural implementation of distractor inhibition theory [68] produce a reaction time benefit by residual activation of the prime target, but of a lower magnitude compared to inhibition in the NP case. In contrast comparable studies usually show larger PP than NP effects [22], [30].

We favor the explanation of the recognition benefits being perceptually caused. This means that any repetition of an object leads to a faster perceptual processing. If the object is repeated identically and was attended in the prime trial, perceptual acceleration is even stronger. This interpretation fits nicely with the results from the EEG study of Behrendt et al. [47]. We therefore postulate the emergence of NP to be a correlate of the selection aspect of selective attention.

## Discussion

In the current study we introduced an experimental approach to disentangle the cognitive processes underlying the negative priming effect in identity priming paradigms. According to our results, positive priming is highly dependent on perceptual similarity, while NP is not. Furthermore we showed that during semantic recognition no NP is observable. We conclude that NP is produced in later stages of a trial, it seems to be a product of target selection or response generation. With a series of three experiments we addressed the question whether NP is produced in the perceptual processing stages of an experimental trial. Experiment 1 focused on the interaction between target color and priming effects. In Experiment 2 the target color was varied trial-wise in an unpredictable order. In this way we reproduced prime-probe pairs that were perceptually identical to the standard conditions, although their experimental condition was different for different target colors. We were thus able to consider an effect being perceptually produced versus occurring during semantic processing of the trial. Experiment 3 went a step further by considering not only the perceptual phase but the recognition of both stimuli that were not identifiable as target and distractor before the recognition response.

Experiment 1 replicated typical priming effects found in voicekey identity negative priming paradigms: A strong acceleration of reactions in TT trials, a weaker acceleration in DD trials, a deceleration in DT trials and a weak deceleration in TD trials. The target color contributed to the reaction times such that responding to red targets was faster than responding to green targets, presumably because of the higher saliency of the color red. Importantly, no interaction between target color and priming effects was observed, providing an adequate basis for the following two experiments which mixed trials with red or green as target color.

Experiment 2 showed results similar to Experiment 1 in trials with no target color change. However, in trials requiring a target color switch, a very interesting pattern arose: While the strong acceleration in TT trials vanished, DT and TD trials showed no difference to their no-switch counterparts even though the display was identical to the no-switch TT condition. The absence of positive priming in TT switch trials strongly suggests that PP is dependent on perceptual processes. The fact that the NP effect in both DT and TD conditions was not affected by a target color switch indicates that NP has no perceptual basis, but that the phenomenon is due to interferences on a semantic level of processing.

Experiment 3 was successful in demonstrating an acceleration of the recognition time in all priming conditions with identical target repetition producing faster reactions than the other three conditions which were statistically indistinguishable. A grouped analysis revealed that the repetition of an object in the same color leads to a highly significant acceleration as compared to an object being repeated in a different color. Experiment 3 showed that before a semantic recognition of the stimuli no slowing is present. Instead, trials showing a repeated object are faster in general.

In summary, we found evidence for PP, unlike NP, to be a perceptual phenomenon. Furthermore, we conclude that NP is not produced during semantic recognition, but is a consequence of selection mechanisms. Thus, even if PP and NP are usually considered together, they are caused by different processes, which has severe theoretical implications. The devotion of PP to perceptual processes is problematic for both retrieval theories as well as distractor inhibition theory because they attempt to explain PP and NP in a single framework. The inclusion of PP in a theoretical account leads the theory away from a simple description.

Distractor inhibition theory hypothesizes the NP effect to be a product of persistent inhibition carried over from the prime trial as a consequence of the selection mechanisms during prime processing [3]. Thus, NP should be most prominent at the beginning of a trial, when the perceptual activation collides with the residual inhibition of the object representation. Distractor inhibition can explain NP being independent of a target color switch in Experiment 2, as it assumes the inhibition to act on semantic representations and not on early perceptual processes. But the inclusion of semantic recognition in the first phase of Experiment 3 did not produce NP as predicted by distractor inhibition, but rather an acceleration also in Dd trials. Even though accelerations in TT trials are much less prominent in the implementation of distractor inhibition theory [31], the theory is in general able to explain accelerations in the recognition phase if a relevant object is repeated by persistent excitation.

Response retrieval theory inherently assumes all effects to be produced primarily during response selection, i.e., the last phase of trial processing. Depending on how literally the statements of response retrieval theory are taken, the temporal determination of PP during recognition is either problematic for the theory or not. On the one hand, response retrieval explicitly links all priming effects to the automatic retrieval of the prime response, which implies no priming effects early in the trial [29]. On the other hand, both retrieval theories lack a description of the assumed slow algorithmic trial processing which might account for early priming effects. Furthermore, retrieval theories do not exclude the involvement of additional processes explicitly which could also be a source of reaction time effects.

Episodic retrieval theory explains the finding that NP is caused by selection mechanisms in a straightforward manner. In case the retrieved episode is incongruent to the current trial, i.e., differing roles of the repeated object, or differing responses, the arising conflicts have to be resolved [8], [39]. This resolution consumes time which results in NP effects. In addition, episodic retrieval theory can well explain the acceleration of the recognition in all priming conditions, as it assumes a recall of the entire prime episode in parallel to recognition. The representation of the repeated object is thus driven both by the percept as well as by the recalled memory, recognition of a repeated object is accelerated as compared to unrelated displays. Concerning the even stronger acceleration in Ts trials, two mechanisms could simultaneously modulate priming in the context of episodic retrieval theory: different encoding strengths of objects according to their relevance, and a modulating effect of perceptual match on retrieval strength.

Future research could address the issue that the design of the present study does not allow us to answer the question whether NP is produced during target or response selection. The reason is a confound of target identity and response inherent to the present voicekey paradigm. This confound has been the subject of a recent debate [29] and can be resolved by an orthogonal variation of response and target identity. This can e.g. be achieved using a comparison task, e.g. [56] instead of the identification task used in the current study.

Summarizing, we have presented a novel approach for splitting trial processing into consecutive stages in order to consider priming effects in these stages individually. In order to establish our paradigm, we started a series of three experiments with a generic identity-NP paradigm and varied only the target color. In the second experiment the target color was randomly altered on each trial. In this way NP trials could be performed with identical stimuli and repetition priming trials with non-identical stimuli. The most interesting result from this experiment is the disappearance of positive priming in case of a color switch, while NP is unaffected by a target color switch. In the third experiment, the target color was revealed only after the stimuli were recognized. This allowed us to separate stimulus recognition and target selection. In the recognition phase, no NP was observed, but all four stimulus repetition conditions led to faster recognition times.
